# Two truncating variants in *FANCC* and breast cancer risk

**DOI:** 10.1038/s41598-019-48804-y

**Published:** 2019-08-29

**Authors:** Thilo Dörk, Paolo Peterlongo, Arto Mannermaa, Manjeet K. Bolla, Qin Wang, Joe Dennis, Thomas Ahearn, Irene L. Andrulis, Hoda Anton-Culver, Volker Arndt, Kristan J. Aronson, Annelie Augustinsson, Laura E. Beane Freeman, Matthias W. Beckmann, Alicia Beeghly-Fadiel, Sabine Behrens, Marina Bermisheva, Carl Blomqvist, Natalia V. Bogdanova, Stig E. Bojesen, Hiltrud Brauch, Hermann Brenner, Barbara Burwinkel, Federico Canzian, Tsun L. Chan, Jenny Chang-Claude, Stephen J. Chanock, Ji-Yeob Choi, Hans Christiansen, Christine L. Clarke, Fergus J. Couch, Kamila Czene, Mary B. Daly, Isabel dos-Santos-Silva, Miriam Dwek, Diana M. Eccles, Arif B. Ekici, Mikael Eriksson, D. Gareth Evans, Peter A. Fasching, Jonine Figueroa, Henrik Flyger, Lin Fritschi, Marike Gabrielson, Manuela Gago-Dominguez, Chi Gao, Susan M. Gapstur, Montserrat García-Closas, José A. García-Sáenz, Mia M. Gaudet, Graham G. Giles, Mark S. Goldberg, David E. Goldgar, Pascal Guénel, Lothar Haeberle, Christopher A. Haiman, Niclas Håkansson, Per Hall, Ute Hamann, Mikael Hartman, Jan Hauke, Alexander Hein, Peter Hillemanns, Frans B. L. Hogervorst, Maartje J. Hooning, John L. Hopper, Tony Howell, Dezheng Huo, Hidemi Ito, Motoki Iwasaki, Anna Jakubowska, Wolfgang Janni, Esther M. John, Audrey Jung, Rudolf Kaaks, Daehee Kang, Pooja Middha Kapoor, Elza Khusnutdinova, Sung-Won Kim, Cari M. Kitahara, Stella Koutros, Peter Kraft, Vessela N. Kristensen, Ava Kwong, Diether Lambrechts, Loic Le Marchand, Jingmei Li, Sara Lindström, Martha Linet, Wing-Yee Lo, Jirong Long, Artitaya Lophatananon, Jan Lubiński, Mehdi Manoochehri, Siranoush Manoukian, Sara Margolin, Elena Martinez, Keitaro Matsuo, Dimitris Mavroudis, Alfons Meindl, Usha Menon, Roger L. Milne, Nur Aishah Mohd Taib, Kenneth Muir, Anna Marie Mulligan, Susan L. Neuhausen, Heli Nevanlinna, Patrick Neven, William G. Newman, Kenneth Offit, Olufunmilayo I. Olopade, Andrew F. Olshan, Janet E. Olson, Håkan Olsson, Sue K. Park, Tjoung-Won Park-Simon, Julian Peto, Dijana Plaseska-Karanfilska, Esther Pohl-Rescigno, Nadege Presneau, Brigitte Rack, Paolo Radice, Muhammad U. Rashid, Gad Rennert, Hedy S. Rennert, Atocha Romero, Matthias Ruebner, Emmanouil Saloustros, Marjanka K. Schmidt, Rita K. Schmutzler, Michael O. Schneider, Minouk J. Schoemaker, Christopher Scott, Chen-Yang Shen, Xiao-Ou Shu, Jacques Simard, Susan Slager, Snezhana Smichkoska, Melissa C. Southey, John J. Spinelli, Jennifer Stone, Harald Surowy, Anthony J. Swerdlow, Rulla M. Tamimi, William J. Tapper, Soo H. Teo, Mary Beth Terry, Amanda E. Toland, Rob A. E. M. Tollenaar, Diana Torres, Gabriela Torres-Mejía, Melissa A. Troester, Thérèse Truong, Shoichiro Tsugane, Michael Untch, Celine M. Vachon, Ans M. W. van den Ouweland, Elke M. van Veen, Joseph Vijai, Camilla Wendt, Alicja Wolk, Jyh-Cherng Yu, Wei Zheng, Argyrios Ziogas, Elad Ziv, ABCTB Investigators, Rosemary Balleine, Robert Baxter, Stephen Braye, Jane Carpenter, Jane Dahlstrom, John Forbes, C. Soon Lee, Deborah Marsh, Adrienne Morey, Nirmala Pathmanathan, Rodney Scott, Peter Simpson, Allan Spigelman, Nicholas Wilcken, Desmond Yip, Nikolajs Zeps, NBCS Collaborators, Anne-Lise Børresen-Dale, Grethe I. Grenaker Alnæs, Kristine K. Sahlberg, Lars Ottestad, Rolf Kåresen, Ellen Schlichting, Marit Muri Holmen, Toril Sauer, Vilde Haakensen, Olav Engebråten, Bjørn Naume, Alexander Fosså, Cecile E. Kiserud, Kristin V. Reinertsen, Åslaug Helland, Margit Riis, Jürgen Geisler, Alison M. Dunning, Paul D. P. Pharoah, Detlev Schindler, Peter Devilee, Douglas F. Easton

**Affiliations:** 1 Gynaecology Research Unit, Hannover Medical School, Hannover, Germany; 2 Genome Diagnostics Program, IFOM - the FIRC Institute of Molecular Oncology, Milan, Italy; 3 Translational Cancer Research Area, University of Eastern Finland, Kuopio, Finland; 4 Institute of Clinical Medicine, Pathology and Forensic Medicine, University of Eastern Finland, Kuopio, Finland; 5 Imaging Center, Department of Clinical Pathology, Kuopio University Hospital, Kuopio, Finland; 6 Centre for Cancer Genetic Epidemiology, Department of Public Health and Primary Care, University of Cambridge, Cambridge, UK; 7 Division of Cancer Epidemiology and Genetics, National Cancer Institute, National Institutes of Health, Department of Health and Human Services, Bethesda, MD USA; 8 Fred A. Litwin Center for Cancer Genetics, Lunenfeld-Tanenbaum Research Institute of Mount Sinai Hospital, Toronto, ON Canada; 9 Department of Molecular Genetics, University of Toronto, Toronto, ON Canada; 10 Department of Epidemiology, Genetic Epidemiology Research Institute, University of California Irvine, Irvine, CA USA; 11 Division of Clinical Epidemiology and Aging Research, C070, German Cancer Research Center (DKFZ), Heidelberg, Germany; 12 Department of Public Health Sciences, and Cancer Research Institute, Queen’s University, Kingston, ON Canada; 13 Department of Cancer Epidemiology, Clinical Sciences, Lund University, Lund, Sweden; 14 Department of Gynecology and Obstetrics, Comprehensive Cancer Center ER-EMN, University Hospital Erlangen, Friedrich-Alexander-University Erlangen-Nuremberg, Erlangen, Germany; 15 Division of Epidemiology, Department of Medicine, Vanderbilt Epidemiology Center, Vanderbilt-Ingram Cancer Center, Vanderbilt University School of Medicine, Nashville, TN USA; 16 Division of Cancer Epidemiology, German Cancer Research Center (DKFZ), Heidelberg, Germany; 17 Institute of Biochemistry and Genetics of the Ufa Federal Research Centre of the Russian Academy of Sciences, Ufa, Russia; 18 Department of Oncology, Helsinki University Hospital, University of Helsinki, Helsinki, Finland; 19 Department of Oncology, Örebro University Hospital, Örebro, Sweden; 20 Department of Radiation Oncology, Hannover Medical School, Hannover, Germany; 21 N.N. Alexandrov Research Institute of Oncology and Medical Radiology, Minsk, Belarus; 22 Copenhagen General Population Study, Herlev and Gentofte Hospital, Copenhagen University Hospital, Herlev, Denmark; 23 Department of Clinical Biochemistry, Herlev and Gentofte Hospital, Copenhagen University Hospital, Herlev, Denmark; 24 Faculty of Health and Medical Sciences, University of Copenhagen, Copenhagen, Denmark; 25 Dr. Margarete Fischer-Bosch-Institute of Clinical Pharmacology, Stuttgart, Germany; 26 University of Tübingen, Tübingen, Germany; 27 German Cancer Consortium (DKTK), German Cancer Research Center (DKFZ), Heidelberg, Germany; 28 Division of Preventive Oncology, German Cancer Research Center (DKFZ) and National Center for Tumor Diseases (NCT), Heidelberg, Germany; 29 Molecular Epidemiology Group, C080, German Cancer Research Center (DKFZ), Heidelberg, Germany; 30 Molecular Biology of Breast Cancer, University Womens Clinic Heidelberg, University of Heidelberg, Heidelberg, Germany; 31 Genomic Epidemiology Group, German Cancer Research Center (DKFZ), Heidelberg, Germany; 32 Hong Kong Hereditary Breast Cancer Family Registry, Cancer Genetics Centre, Happy Valley, Hong Kong; 33 Department of Pathology, Hong Kong Sanatorium and Hospital, Happy Valley, Hong Kong; 34 Cancer Epidemiology Group, University Cancer Center Hamburg (UCCH), University Medical Center Hamburg-Eppendorf, Hamburg, Germany; 35 Department of Biomedical Sciences, Seoul National University Graduate School, Seoul, Korea; 36 Cancer Research Institute, Seoul National University, Seoul, Korea; 37 Westmead Institute for Medical Research, University of Sydney, Sydney, New South Wales Australia; 38 Department of Laboratory Medicine and Pathology, Mayo Clinic, Rochester, MN USA; 39 Department of Medical Epidemiology and Biostatistics, Karolinska Institutet, Stockholm, Sweden; 40 Department of Clinical Genetics, Fox Chase Cancer Center, Philadelphia, PA USA; 41 Department of Non-Communicable Disease Epidemiology, London School of Hygiene and Tropical Medicine, London, UK; 42 Department of Biomedical Sciences, Faculty of Science and Technology, University of Westminster, London, UK; 43 Cancer Sciences Academic Unit, Faculty of Medicine, University of Southampton, Southampton, UK; 44 Institute of Human Genetics, University Hospital Erlangen, Friedrich-Alexander University Erlangen-Nuremberg, Comprehensive Cancer Center Erlangen-EMN, Erlangen, Germany; 45 Division of Evolution and Genomic Sciences, School of Biological Sciences, Faculty of Biology, Medicine and Health, University of Manchester, Manchester Academic Health Science Centre, Manchester, UK; 46 Manchester Centre for Genomic Medicine, St Mary’s Hospital, Manchester University Hospitals NHS Foundation Trust, Manchester Academic Health Science Centre, Manchester, UK; 47 David Geffen School of Medicine, Department of Medicine Division of Hematology and Oncology, University of California at Los Angeles, Los Angeles, CA USA; 48 Usher Institute of Population Health Sciences and Informatics, The University of Edinburgh Medical School, Edinburgh, UK; 49 Cancer Research UK Edinburgh Centre, Edinburgh, UK; 50 Department of Breast Surgery, Herlev and Gentofte Hospital, Copenhagen University Hospital, Herlev, Denmark; 51 School of Public Health, Curtin University, Perth, Western Australia Australia; 52 Genomic Medicine Group, Galician Foundation of Genomic Medicine, Instituto de Investigación Sanitaria de Santiago de Compostela (IDIS), Complejo Hospitalario Universitario de Santiago, SERGAS, Santiago de Compostela, Spain; 53 Moores Cancer Center, University of California San Diego, La Jolla, CA USA; 54 Program in Genetic Epidemiology and Statistical Genetics, Harvard T.H. Chan School of Public Health, Boston, MA USA; 55 Department of Epidemiology, Harvard T.H. Chan School of Public Health, Boston, MA USA; 56 Behavioral and Epidemiology Research Group, American Cancer Society, Atlanta, GA USA; 57 Division of Genetics and Epidemiology, Institute of Cancer Research, London, UK; 58 Medical Oncology Department, Hospital Clínico San Carlos, Instituto de Investigación Sanitaria San Carlos (IdISSC), Centro Investigación Biomédica en Red de Cáncer (CIBERONC), Madrid, Spain; 59 Cancer Epidemiology Division, Cancer Council Victoria, Melbourne, Victoria, Australia; 60 Centre for Epidemiology and Biostatistics, Melbourne School of Population and Global Health, The University of Melbourne, Melbourne, Victoria, Australia; 61 Department of Epidemiology and Preventive Medicine, Monash University, Melbourne, Victoria, Australia; 62 Department of Medicine, McGill University, Montréal, QC Canada; 63 Division of Clinical Epidemiology, Royal Victoria Hospital, McGill University, Montréal, QC Canada; 64 Department of Dermatology, Huntsman Cancer Institute, University of Utah School of Medicine, Salt Lake City, UT USA; 65 Cancer & Environment Group, Center for Research in Epidemiology and Population Health (CESP), INSERM, University Paris-Sud, University Paris-Saclay, Villejuif, France; 66 Department of Gynaecology and Obstetrics, University Hospital Erlangen, Friedrich-Alexander University Erlangen-Nuremberg, Comprehensive Cancer Center Erlangen-EMN, Erlangen, Germany; 67 Department of Preventive Medicine, Keck School of Medicine, University of Southern California, Los Angeles, CA USA; 68 Institute of Environmental Medicine, Karolinska Institutet, Stockholm, Sweden; 69 Department of Oncology, Södersjukhuset, Stockholm, Sweden; 70 Molecular Genetics of Breast Cancer, German Cancer Research Center (DKFZ), Heidelberg, Germany; 71 Saw Swee Hock School of Public Health, National University of Singapore, Singapore, Singapore; 72 Department of Surgery, National University Health System, Singapore, Singapore; 73 Center for Familial Breast and Ovarian Cancer, Faculty of Medicine and University Hospital Cologne, University of Cologne, Cologne, Germany; 74 Center for Molecular Medicine Cologne (CMMC), Faculty of Medicine and University Hospital Cologne, University of Cologne, Cologne, Germany; 75 Center for Integrated Oncology (CIO), Faculty of Medicine and University Hospital Cologne, University of Cologne, Cologne, Germany; 76 Family Cancer Clinic, The Netherlands Cancer Institute - Antoni van Leeuwenhoek hospital, Amsterdam, The Netherlands; 77 Department of Medical Oncology, Family Cancer Clinic, Erasmus MC Cancer Institute, Rotterdam, The Netherlands; 78 Division of Cancer Sciences, University of Manchester, Manchester, UK; 79 Center for Clinical Cancer Genetics, The University of Chicago, Chicago, IL USA; 80 Division of Cancer Epidemiology and Prevention, Aichi Cancer Center Research Institute, Nagoya, Japan; 81 Division of Cancer Epidemiology, Nagoya University Graduate School of Medicine, Nagoya, Japan; 82 Division of Epidemiology, Center for Public Health Sciences, National Cancer Center, Tokyo, Japan; 83 Department of Genetics and Pathology, Pomeranian Medical University, Szczecin, Poland; 84 Independent Laboratory of Molecular Biology and Genetic Diagnostics, Pomeranian Medical University, Szczecin, Poland; 85 Department of Gynaecology and Obstetrics, University Hospital Ulm, Ulm, Germany; 86 Department of Medicine, Division of Oncology, Stanford Cancer Institute, Stanford University School of Medicine, Stanford, CA USA; 87 Department of Preventive Medicine, Seoul National University College of Medicine, Seoul, Korea; 88 Faculty of Medicine, University of Heidelberg, Heidelberg, Germany; 89 Department of Genetics and Fundamental Medicine, Bashkir State University, Ufa, Russia; 90 Department of Surgery, Daerim Saint Mary’s Hospital, Seoul, Korea; 91 Radiation Epidemiology Branch, Division of Cancer Epidemiology and Genetics, National Cancer Institute, Bethesda, MD USA; 92 Department of Cancer Genetics, Institute for Cancer Research, Oslo University Hospital-Radiumhospitalet, Oslo, Norway; 93 Institute of Clinical Medicine, Faculty of Medicine, University of Oslo, Oslo, Norway; 94 Department of Surgery, The University of Hong Kong, Pok Fu Lam, Hong Kong; 95 Department of Surgery, Hong Kong Sanatorium and Hospital, Happy Valley, Hong Kong; 96 VIB Center for Cancer Biology, VIB, Leuven, Belgium; 97 Laboratory for Translational Genetics, Department of Human Genetics, University of Leuven, Leuven, Belgium; 98 Epidemiology Program, University of Hawaii Cancer Center, Honolulu, HI USA; 99 Human Genetics Division, Genome Institute of Singapore, Singapore, Singapore; 100 Department of Epidemiology, University of Washington School of Public Health, Seattle, WA USA; 101 Public Health Sciences Division, Fred Hutchinson Cancer Research Center, Seattle, WA USA; 102 Division of Health Sciences, Warwick Medical School, University of Warwick, Coventry, UK; 103 Division of Population Health, Health Services Research and Primary Care, School of Health Sciences, Faculty of Biology, Medicine and Health, The University of Manchester, Manchester, UK; 104 Unit of Medical Genetics, Department of Medical Oncology and Hematology, Fondazione IRCCS Istituto Nazionale dei Tumori di Milano, Milan, Italy; 105 Department of Clinical Science and Education, Södersjukhuset, Karolinska Institutet, Stockholm, Sweden; 106 Department of Family Medicine and Public Health, University of California San Diego, La Jolla, CA USA; 107 Department of Medical Oncology, University Hospital of Heraklion, Heraklion, Greece; 108 Department of Gynecology and Obstetrics, Ludwig Maximilian University of Munich, Munich, Germany; 109 MRC Clinical Trials Unit at UCL, Institute of Clinical Trials & Methodology, University College London, London, UK; 110 Precision Medicine, School of Clinical Sciences at Monash Health, Monash University, Clayton, Victoria, Australia; 111 Breast Cancer Research Unit, UM Cancer Research Institute, University of Malaya Medical Centre, Kuala Lumpur, Malaysia; 112 Department of Laboratory Medicine and Pathobiology, University of Toronto, Toronto, ON Canada; 113 Laboratory Medicine Program, University Health Network, Toronto, ON Canada; 114 Department of Population Sciences, Beckman Research Institute of City of Hope, Duarte, CA USA; 115 Department of Obstetrics and Gynecology, Helsinki University Hospital, University of Helsinki, Helsinki, Finland; 116 Leuven Multidisciplinary Breast Center, Department of Oncology, Leuven Cancer Institute, University Hospitals Leuven, Leuven, Belgium; 117 Clinical Genetics Research Lab, Department of Cancer Biology and Genetics, Memorial Sloan-Kettering Cancer Center, New York, NY USA; 118 Clinical Genetics Service, Department of Medicine, Memorial Sloan-Kettering Cancer Center, New York, NY USA; 119 Department of Epidemiology, Gillings School of Global Public Health and UNC Lineberger Comprehensive Cancer Center, University of North Carolina at Chapel Hill, Chapel Hill, NC USA; 120 Department of Health Sciences Research, Mayo Clinic, Rochester, MN USA; 121 Research Centre for Genetic Engineering and Biotechnology ‘Georgi D. Efremov’, Macedonian Academy of Sciences and Arts, Skopje, Republic of Macedonia; 122 Unit of Molecular Bases of Genetic Risk and Genetic Testing, Department of Research, Fondazione IRCCS Istituto Nazionale dei Tumori (INT), Milan, Italy; 123 Department of Basic Sciences, Shaukat Khanum Memorial Cancer Hospital and Research Centre (SKMCH & RC), Lahore, Pakistan; 124 Clalit National Cancer Control Center, Carmel Medical Center and Technion Faculty of Medicine, Haifa, Israel; 125 Medical Oncology Department, Hospital Universitario Puerta de Hierro, Madrid, Spain; 126 Department of Oncology, University Hospital of Larissa, Larissa, Greece; 127 Division of Molecular Pathology, The Netherlands Cancer Institute - Antoni van Leeuwenhoek Hospital, Amsterdam, The Netherlands; 128 Division of Psychosocial Research and Epidemiology, The Netherlands Cancer Institute - Antoni van Leeuwenhoek hospital, Amsterdam, The Netherlands; 129 Division of Genetics and Epidemiology, The Institute of Cancer Research, London, UK; 130 Institute of Biomedical Sciences, Academia Sinica, Taipei, Taiwan; 131 School of Public Health, China Medical University, Taichung, Taiwan; 132 Genomics Center, Centre Hospitalier Universitaire de Québec – Université Laval Research Center, Québec City, QC Canada; 133 Ss. Cyril and Methodius University in Skopje, Medical Faculty, University Clinic of Radiotherapy and Oncology, Skopje, Republic of Macedonia; 134 Department of Clinical Pathology, The University of Melbourne, Melbourne, Victoria Australia; 135 Population Oncology, BC Cancer, Vancouver, BC Canada; 136 School of Population and Public Health, University of British Columbia, Vancouver, BC Canada; 137 The Curtin UWA Centre for Genetic Origins of Health and Disease, Curtin University and University of Western Australia, Perth, Western Australia Australia; 138 Division of Breast Cancer Research, The Institute of Cancer Research, London, UK; 139 Channing Division of Network Medicine, Department of Medicine, Brigham and Women’s Hospital and Harvard Medical School, Boston, MA USA; 140 Faculty of Medicine, University of Southampton, Southampton, UK; 141 Cancer Research Malaysia, Subang Jaya, Selangor Malaysia; 142 Department of Epidemiology, Mailman School of Public Health, Columbia University, New York, NY USA; 143 Department of Cancer Biology and Genetics, The Ohio State University, Columbus, OH USA; 144 Department of Surgery, Leiden University Medical Center, Leiden, The Netherlands; 145 Institute of Human Genetics, Pontificia Universidad Javeriana, Bogota, Colombia; 146 Center for Population Health Research, National Institute of Public Health, Mexico, Mexico; 147 Center for Public Health Sciences, National Cancer Center, Tokyo, Japan; 148 Department of Gynecology and Obstetrics, Helios Clinics Berlin-Buch, Berlin, Germany; 149 Department of Health Science Research, Division of Epidemiology, Mayo Clinic, Rochester, MN USA; 150 Department of Clinical Genetics, Erasmus University Medical Center, Rotterdam, The Netherlands; 151 Department of Surgical Sciences, Uppsala University, Uppsala, Sweden; 152 Department of Surgery, Tri-Service General Hospital, National Defense Medical Center, Taipei, Taiwan; 153 Department of Medicine, Institute for Human Genetics, UCSF Helen Diller Family Comprehensive Cancer Center, University of California San Francisco, San Francisco, CA USA; 154 Westmead Institute for Medical Research, University of Sydney, Sydney, New South Wales, Australia; 155 Department of Research, Vestre Viken Hospital, Drammen, Norway; 156 iFIT Cluster of Excellence, University of Tübingen, Tübingen, Germany; 157 Section for Breast- and Endocrine Surgery, Department of Cancer, Division of Surgery, Cancer and Transplantation Medicine, Oslo University Hospital-Ullevål, Oslo, Norway; 158 Department of Radiology and Nuclear Medicine, Oslo University Hospital, Oslo, Norway; 159 Department of Pathology, Akershus University Hospital, Lørenskog, Norway; 160 Department of Tumor Biology, Institute for Cancer Research, Oslo University Hospital, Oslo, Norway; 161 Department of Oncology, Division of Surgery, Cancer and Transplantation Medicine, Oslo University Hospital-Radiumhospitalet, Oslo, Norway; 162 National Advisory Unit on Late Effects after Cancer Treatment, Oslo University Hospital-Radiumhospitalet, Oslo, Norway; 163 Department of Oncology, Akershus University Hospital, Lørenskog, Norway; 164 Breast Cancer Research Consortium, Oslo University Hospital, Oslo, Norway; 165 Centre for Cancer Genetic Epidemiology, Department of Oncology, University of Cambridge, Cambridge, UK; 166 Institute of Human Genetics, Biocenter, University of Würzburg, Würzburg, Germany; 167 Department of Pathology, Leiden University Medical Center, Leiden, The Netherlands; 168 Department of Human Genetics, Leiden University Medical Center, Leiden, The Netherlands; 169 Pathology West ICPMR, Westmead, Sydney, NSW Australia; 170 Kolling Institute of Medical Research, University of Sydney, Royal North Shore Hospital, Sydney, NSW Australia; 171 Pathology North, John Hunter Hospital, Newcastle, NSW Australia; 172 Department of Anatomical Pathology, ACT Pathology, Canberra Hospital, Canberra, ACT Australia; 173 ANU Medical School, Australian National University, Canberra, ACT Australia; 174 Department of Surgical Oncology, Calvary Mater Newcastle Hospital, Australian New Zealand Breast Cancer Trials Group, and School of Medicine and Public Health, University of Newcastle, Newcastle, NSW Australia; 175 School of Science and Health, The University of Western Sydney, Sydney, NSW Australia; 176 Hormones and Cancer Group, Kolling Institute of Medical Research, Royal North Shore Hospital, University of Sydney, Sydney, NSW Australia; 177 SydPath St Vincent’s Hospital, Sydney, NSW Australia; 178 Department of Tissue Pathology and Diagnostic Oncology, Pathology West, Westmead Breast Cancer Institute, Westmead Hospital, Sydney, NSW Australia; 179 Centre for Information Based Medicine, Hunter Medical Research Institute, Newcastle, NSW Australia; 180 Priority Research Centre for Cancer, School of Biomedical Sciences and Pharmacy, Faculty of Health, University of Newcastle, Newcastle, NSW Australia; 181 The University of Queensland: UQ Centre for Clinical Research and School of Medicine, Brisbane, QLD Australia; 182 Hereditary Cancer Clinic, St Vincent’s Hospital, The Kinghorn Cancer Centre, Sydney, NSW Australia; 183 Crown Princess Mary Cancer Centre, Westmead Hospital, Sydney, NSW Australia; 184 Sydney Medical School - Westmead, University of Sydney, Sydney, NSW Australia; 185 Department of Medical Oncology, The Canberra Hospital, Canberra, ACT Australia; 186 St John of God Perth Northern Hospitals, Perth, WA Australia

**Keywords:** Oncology, Risk factors

## Abstract

Fanconi anemia (FA) is a genetically heterogeneous disorder with 22 disease-causing genes reported to date. In some FA genes, monoallelic mutations have been found to be associated with breast cancer risk, while the risk associations of others remain unknown. The gene for FA type C, *FANCC*, has been proposed as a breast cancer susceptibility gene based on epidemiological and sequencing studies. We used the Oncoarray project to genotype two truncating *FANCC* variants (p.R185X and p.R548X) in 64,760 breast cancer cases and 49,793 controls of European descent. *FANCC* mutations were observed in 25 cases (14 with p.R185X, 11 with p.R548X) and 26 controls (18 with p.R185X, 8 with p.R548X). There was no evidence of an association with the risk of breast cancer, neither overall (odds ratio 0.77, 95%CI 0.44–1.33, p = 0.4) nor by histology, hormone receptor status, age or family history. We conclude that the breast cancer risk association of these two *FANCC* variants, if any, is much smaller than for *BRCA1*, *BRCA2* or *PALB2* mutations. If this applies to all truncating variants in *FANCC* it would suggest there are differences between FA genes in their roles on breast cancer risk and demonstrates the merit of large consortia for clarifying risk associations of rare variants.

## Introduction

Fanconi Anemia (FA) is a rare recessively inherited disorder characterized by congenital malformations, progressive bone marrow failure and predisposition to cancer. Twenty-two different FA causative genes have now been identified whose products act in concert to mediate DNA interstrand crosslink repair^1–3^. At least seven of them (*BRCA2*/*FANCD2*, *PALB2*/*FANCN*, *RAD51C*/*FANCO*, *RAD51*/*FANCR*, *BRCA1*/*FANCS*, *XRCC2*/*FANCU*, and *RFWD3*/*FANCW*) are involved in different stages of homology-directed recombinational DNA repair (HRR), a pathway for error-free maintenance of the genome during replication and after DNA damage. A number of FA genes (including *BRCA1*/*FANCS*, *BRCA2*/*FANCD1* and *PALB2*/*FANCN*) have been shown to be breast cancer susceptibility genes^3^. The products of *BRCA1*, *BRCA2*, and *PALB2* are central to early stages of HRR. Further interactors in this pathway, in particular *BRIP1*/*FANCJ*, mainly have been linked to ovarian cancer risk^4,5^. It is less known to what extent other FA gene products may play a role in the inherited component of breast cancer susceptibility. Few of these other FA genes have been tested for mutations in relatively small breast cancer case-control studies, thus far^6–9^.

Early studies suggested that blood relatives of FA patients show an increased risk of breast cancer, although these findings have not been corroborated in a replication study and could not assess distinct FA complementation groups due to lack of genetic information at that time^10–13^. After FA was stratified into subsets defined by complementation assays, an increased risk of breast cancer was attributed to heterozygous carriers of *FANCC* mutations^13^. Historically, this was the first of the FA genes to be identified and accounts for 8–15% of FA cases^14–16^. More recently, *FANCC* has been suggested as a candidate breast cancer susceptibility gene in an exome sequencing study of 33 familial breast cancer cases and extension to another 438 cases^17^. However, the evidence for an association between *FANCC* and breast cancer risk is limited by the low prevalence of mutations^17,18^, and much larger numbers of individuals are needed to provide sufficient power to detect associations of plausible magnitude^19^.

In the present study, we genotyped two truncating variants of *FANCC* (p.R185X and p.R548X) using the Oncoarray (see Methods) in 64,760 female breast cancer cases and 49,793 female population controls of European descent. Both mutations are disease-causing in European FA patients and are recurrent in the FA mutation database^20^.

## Results

We identified the truncating *FANCC* variants p.R185X (rs121917783) and p.R548X (rs104886457) in 40 of 153,899 individuals and 20 of 153,904 individuals, respectively. All mutation carriers were heterozygotes. Carrier distributions per study and intensity cluster plots for Europeans (which included the majority of mutation carriers) are shown in Supplementary Table 1 and Supplementary Fig. 1, respectively. Since the majority of carriers were women of European ancestry, we restricted the subsequent case-control association analysis to participants from this population. Logistic regression analyses were adjusted for study and 15 principal components^21^.

In Europeans, the two *FANCC* variants were observed in 25/64,760 cases (14 with p.R185X, 11 with p.R548X) and in 26/49,793 controls (18 with p.R185X, 8 with p.R548X). There was no evidence of association between the *FANCC* variants and breast cancer risk, either for carriers of both variants combined (OR 0.77, 95%CI 0.44–1.33, p = 0.35), or for either variant individually (Table 1). Similarly, we found no evidence for an association with estrogen receptor (ER)-negative (OR 0.91, 0.35–2.37) or ER-positive (OR 0.67, 0.37–1.28) disease, nor for subsets of disease defined by age at diagnosis (<50 years), bilaterality, family history, histological morphology, grade or nodal status (Table 2).

**Table 1:** Overall analysis of *FANCC* variants p.R158X and p.R548X.

Mutation	Cases	Controls	Odds Ratio (95% CI)	p
p.R158X	14/64,778	18/49,810	0.64 (0.32; 1.29)	0.215
p.R548X	11/64,788	8/49,816	1.03 (0.41; 2.56)	0.942
All *FANCC*	**25/64**,**760**	**26/49**,**793**	**0.77** (**0.44**; **1.33**)	**0.345**

Association analyses of *FANCC* variants p.R158X and p.R548X with overall breast cancer risk. Results are given as odds ratios (OR) with 95% confidence interval (CI) and p-value (p).

**Table 2:** Analysis of *FANCC* variants (p.R158X and p.R548X combined) by tumour subtype.

Stratum	Cases	Odds Ratio (95% CI)	p
ER-negative	5/10,124	0.91 (0.35; 2.37)	0.845
ER-positive	14/40,855	0.67 (0.37; 1.28)	0.223
TNBC	2/4,126	0.89 (0.21; 3.77)	0.877
Ductal	6/36,695	0.33 (0.13; 0.80)	0.014
Lobular	4/6,842	1.27 (0.43; 3.69)	0.665
High grade	3/14,582	0.39 (0.12; 1.31)	0.129
Node-positive	1/15,937	0.14 (0.02; 1.00)	0.050
Familial	7/9,720	1.01 (0.43; 2.35)	0.988
Premenopausal	12/22,232	1.09 (0.55; 2.16)	0.814
Bilateral	0/2,741	—	0.645

Association analyses of *FANCC* variants p.R158X and p.R548X with breast cancer risk for subgroups. Results are given as odds ratios (OR) with 95% confidence interval (CI) and p-value (p). Cases in subgroups were compared to the frequency 26/ 49,793 for all controls (derived from Table 1). Familial cases were defined as those with a first-degree family history of breast cancer; premenopausal cases were those with age at diagnosis <50 years. ER, estrogen-receptor; TNBC, triple-negative breast cancer.

For comparison, we also analysed the *PALB2*/FANCN*p.R414X truncating variant that was genotyped in parallel on the same array. This variant was detected in 22/64,780 cases and 3/49,825 controls and was significantly associated with risk of breast cancer (OR 5.89, 95%CI 1.76–19.74, p = 0.004). The variant carriers were markedly enriched among cases with ER-negative tumours (p = 9.4 × 10^−6^; p_diff_ = 0.0006 in a log-likelihood ratio test) and specifically triple-negative breast tumours (p = 3.8 × 10^−7^; p_diff_ = 0.0001). The p.R414X truncating variant was also associated with ductal morphology, a positive first-degree family history of breast cancer, early age at diagnosis (<50 years), and low-differentiated tumours (grade 3) (Suppl. Table 1). Hence, by contrast with the two tested *FANCC* variants, p.R185X and p.R548X, the *FANCN*/*PALB2* variant p.R414X was strongly associated with overall and with ER-negative disease under the same genotyping and analysis conditions.

## Discussion

Functional defects of DNA repair are a hallmark of genomic instability syndromes as well as of carcinogenesis. FA is a genome instability and cancer prone disorder that has been investigated for breast cancer predisposition in homozygotes and heterozygotes for more than three decades^11,12^. Monoallelic mutations in five FA genes (*BRCA1*, *BRCA2*, *PALB2*, *RAD51C*, *BRIP1*) have now been confirmed to predispose to breast or ovarian cancer while biallelic mutations in these genes cause FA^3^. However, the role of the FA genes most commonly mutated, *FANCA* and *FANCC*, in the risk of developing breast cancer has remained uncertain. Epidemiological and segregation studies have provided some evidence of an increased breast cancer risk for grandmothers of FA patients, particularly those who carry the *FANCC* mutation^13^.

A previous sequencing study of Australian multiple-case breast cancer families had identified truncating variants in *FANCC* in 3 of 438 multiple-case breast cancer families but in none of 464 healthy controls, suggestive of a predisposing role for *FANCC* variants in breast cancer^17^. One of these variants, p.R185X, was also screened in our study. p.R185X was first reported shortly after the identification of the *FANCC* gene, and thus is one of the earliest recognized FA-causing mutations. Although representing an apparent nonsense mutation in exon 6, it also results in exon 6 being spliced out of a proportion of transcripts, suggesting this variant may alter splice site selection, with the aberrant transcript retaining the reading frame^22^. p.R548X, also an early-detected *FANCC* truncating variant^23^, is an authentic stop mutation in exon 14, and although in the last exon, it proved to be clearly pathogenic for FA^24^.

The fact that these two disease-causing variants have been frequently observed in European patients with FA^20^ prompted us to investigate their association with breast cancer in a large case-control study. However, we did not observe a significant difference between their frequency among breast cancer cases and controls. The upper 95% confidence limit was 1.33, thus excluding a two-fold or greater increase in risk found for moderate- or high-penetrance alleles in predisposition genes such as *CHEK2* and *ATM*. Moreover, we found no evidence of association in subgroups defined by earlier age at onset, a positive family history of breast cancer, bilateral occurrence, or defined tumor parameters (histology, grade or hormone receptor status). However, confidence intervals for those estimates for subsets were wider as numbers were small – in particular we could not rule out a 2-fold increased risk for ER-negative or triple-negative breast cancer.

In contrast, we observed a clear association between the *PALB2*/*FANCN* variant p.R414X and breast cancer risk. *PALB2* is an established breast cancer susceptibility gene, and the investigated mutation p.R414X^25^ occurred at a similar frequency to the tested *FANCC* mutations. The observed six-fold enrichment of p.R414X in breast cancer patients is in line with previous findings for other *PALB2* founder mutations^26–28^ and in the upper range of the overall mutational effect size in *PALB2* case-control sequencing studies^29,30^. We confirmed stronger associations with ER-negative breast cancer, with familial breast cancer and with a high tumor grade^31^. While genotyping arrays such as the Oncoarray are primarily used for evaluating common variants, these data confirm that the array provides a robust platform for evaluating even very rare alleles.

Although *PALB2* and *FANCC* are both FA genes, their products exert different roles in the recognition and repair of DNA damage. FANCC is a component of the FA core complex which is thought to recognize an inter-strand crosslink. FANCL, an E3 ubiquitin ligase in the core complex, ubiquitinates FANCI and FANCD2. After many nuclease and translesion polymerase steps, a DNA double stranded intermediate is formed and its repair requires proteins from the homology-directed repair pathway, including FANCD1/BRCA2 and FANCN/PALB2. While truncating variants in *BRCA2* and *PALB2* confer a substantial risk of breast cancer, our study suggests that truncating *FANCC* variants do not confer a comparable risk. It is possible that members of the FA core complex that act upstream of HRR are less relevant for breast cancer due to their more specialized function in the repair of crosslinks while BRCA1, BRCA2, and PALB2 function more globally at DNA double-strand breaks. On the other hand, there is some evidence that truncating mutations in another gene involved in the early detection of intra-strand crosslinks, *FANCM*, are associated with both breast and ovarian cancer risk^32–34^, though *FANCM* is part of an anchor complex rather than the FA core complex and is not considered a classical FA gene^35,36^. It is also possible that the two prototype *FANCC* truncating variants analysed here, despite being FA-causing, have reduced penetrance for breast cancer due to some residual function, and other particular *FANCC* variants may confer a more substantial risk. More work will be required to clarify the role of each FA core complex member for breast cancer susceptibility.

In conclusion, our study findings suggest important differences between FA genes, indicating that truncating variants in *FANCC* do not confer a high overall risk of breast cancer unlike *PALB2*, *BRCA1* and *BRCA2*. Our study does not exclude a role of monoallelic *FANCC* variants as low-penetrance alleles for breast cancer or as a genetic risk factor for certain breast cancer subgroups. Very large datasets, such as those generated through the BCAC, are critical to evaluate such rare mutations.

## Methods

### Patients

A total of 87 studies from the Breast Cancer Association Consortium (BCAC), of which 78 were case-control studies (some nested within prospective cohort studies) and 9 were case-only studies, contributed data as summarized in Supplementary Table 1. All studies provided data on disease status and age at diagnosis/observation, and the majority provided information on clinico-pathological and epidemiological factors, which have been curated and incorporated into the BCAC database (version 6). All participating studies were approved by their appropriate ethics review boards and all subjects provided informed consent. A list of the ethics review boards by study is provided in Supplementary Table 3.

### Genotyping

The Illumina OncoArray design and genotyping procedure have been described previously^21,37^. In brief, approximately 72,000 variants were selected, among others, for inclusion on the array specifically for their potential relevance to breast cancer, based on prior evidence of association with overall or subtype-specific disease, with breast density or with breast tissue specific gene expression. After genotype calling and quality control of the cluster file, variants with a call rate <95% in any consortium, not in Hardy-Weinberg equilibrium (P < 10^−7^ in controls or P < 10^−12^ in cases) or with concordance <98% among 5,280 duplicate pairs were excluded. We also excluded samples with extreme heterozygosity (>4.89 standard deviations [SD] from the mean for the respective ethnicity). The final dataset, before restriction based on ethnicity, consisted of 153,673 samples of which 89,733 were cases and 63,940 were controls.

### Statistical analyses

Per-allele odds ratios and 95% confidence intervals were generated using logistic regression with adjustment for principal components and study. Principal component analysis was performed using data for 33,661 uncorrelated SNPs (which included 2,318 markers of continental ancestry) with a MAF ≥ 0.05 and maximum correlation of 0.1, using purpose-written PCcalc software (written by Jonathan Tyrer and available at http://ccge.medschl.cam.ac.uk/software/pccalc/).

We also estimated subtype-specific per-allele ORs after restricting the cases by hormone receptor and/or HER2/neu status, by tumor grade, by ductal or lobular morphology, by nodal status, by bilateral occurrence of the tumor, by early diagnosis (<50 years), and by first-degree family history of breast cancer, using available BCAC data for the cases. Since we analysed 3 variants across 10 subgroups, a two-sided p-value ≤ 0.016 for the overall analyses and a two-sided p-value ≤ 0.0016 for the subgroup analyses were considered nominally significant.

### Ethical approval

All experimental protocols were approved by the respective ethical institutions of participating BCAC centers. The study was carried out in accordance with the Declaration of Helsinki, and informed consent was obtained from all study participants.

## Supplementary information

Supplemental Info

## Data Availability

The genotyping results from the Oncoarray are available in the dbGAP repository. The *FANCC* variants analysed in the current study are deposited in the NCBI SNP database as rs121917783 and rs104886457. The datasets analysed during the current study are available from the corresponding author upon reasonable request and with permission of the Data Access Committee of the Breast Cancer Association Consortium.

## References

[1] Ceccaldi R, Sarangi P, D’Andrea AD (2016). The Fanconi anaemia pathway: new players and new functions. Nat. Rev. Mol. Cell Biol..

[2] Knies K (2017). Biallelic mutations in the ubiquitin ligase RFWD3 cause Fanconi anemia. J. Clin. Invest..

[3] Nalepa G, Clapp DW (2018). Fanconi anaemia and cancer: an intricate relationship. Nat. Rev. Cancer.

[4] Ramus SJ (2015). Germline Mutations in the BRIP1, BARD1, PALB2, and NBN Genes in Women With Ovarian Cancer. J. Natl. Cancer Inst..

[5] Easton DF (2016). No evidence that protein truncating variants in BRIP1 are associated with breast cancer risk: implications for gene panel testing. J. Med. Genet..

[6] García MJ (2009). Mutational analysis of FANCL, FANCM and the recently identified FANCI suggests that among the 13 known Fanconi Anemia genes, only FANCD1/BRCA2 plays a major role in high-risk breast cancer predisposition. Carcinogenesis.

[7] Bakker JL (2013). Analysis of the novel fanconi anemia gene SLX4/FANCP in familial breast cancer cases. Hum. Mutat..

[8] Osorio A (2013). Evaluation of rare variants in the new fanconi anemia gene ERCC4 (FANCQ) as familial breast/ovarian cancer susceptibility alleles. Hum. Mutat..

[9] Lhota F (2016). Hereditary truncating mutations of DNA repair and other genes in BRCA1/BRCA2/PALB2-negatively tested breast cancer patients. Clin. Genet..

[10] Swift M (1971). Fanconi’s anaemia in the genetics of neoplasia. Nature.

[11] Swift M, Caldwell RJ, Chase C (1980). Reassessment of cancer predisposition of Fanconi anemia heterozygotes. J. Natl. Cancer Inst..

[12] Jacobs P, Karabus C (1984). Fanconi’s anemia. A family study with 20-year follow-up including associated breast pathology. Cancer.

[13] Berwick M (2007). Genetic heterogeneity among Fanconi anemia heterozygotes and risk of cancer. Cancer Res..

[14] Strathdee CA (1992). Cloning of cDNAs for Fanconi’s anaemia by functional complementation. Nature.

[15] Gibson RA (1994). Genetic mapping of the FACC gene and linkage analysis in Fanconi anaemia families. J. Med. Genet..

[16] Verlander PC (1994). Mutation analysis of the Fanconi anemia gene FACC. Am. J. Hum. Genet..

[17] Thompson ER (2012). Exome sequencing identifies rare deleterious mutations in DNA repair genes FANCC and BLM as potential breast cancer susceptibility alleles. PLoS Genet..

[18] Seal S (2003). Evaluation of Fanconi Anemia genes in familial breast cancer predisposition. Cancer Res..

[19] Ellis NA, Offit K (2012). Heterozygous mutations in DNA repair genes and hereditary breast cancer: a question of power. PLoS Genet..

[20] Fanconi anemia mutation database, http://www2.rockefeller.edu/fanconi/.

[21] Michailidou, K. *et al*. Association analysis identifies 65 new breast cancer risk loci. *Nature* **551**, 92–94 (2017). 10.1038/nature24284 PMC5798588 29059683

[22] Gibson RA (1993). A nonsense mutation and exon skipping in the Fanconi anaemia group C gene. Hum Mol Genet..

[23] Murer-Orlando, M., Llerena, J. C. Jr. & Birjandi, F. FACC gene mutations and early prenatal diagnosis of Fanconi’s anaemia. *Lancet*. p. 686 (1993). 10.1016/0140-6736(93)91800-2 8103176

[24] Lo ten Foe JR (1996). Sequence variations in the Fanconi anaemia gene, FAC: pathogenicity of 1806insA and R548X and recognition of D195V as a polymorphic variant. Hum Genet..

[25] Bogdanova N (2011). PALB2 mutations in German and Russian patients with bilateral breast cancer. Breast Cancer Res. Treat..

[26] Erkko H (2007). A recurrent mutation in PALB2 in Finnish cancer families. Nature.

[27] Southey MC (2010). A PALB2 mutation associated with high risk of breast cancer. Breast Cancer Res..

[28] Noskowicz M (2014). Prevalence of PALB2 mutation c.509_510delGA in unselected breast cancer patients from Central and Eastern Europe. Fam. Cancer.

[29] Rahman N (2007). PALB2, which encodes a BRCA2-interacting protein, is a breast cancer susceptibility gene. Nat. Genet..

[30] Tischkowitz M (2012). Rare germline mutations in PALB2 and breast cancer risk: a population-based study. Hum. Mutat..

[31] Heikkinen T (2009). The breast cancer susceptibility mutation PALB2 1592delT is associated with an aggressive tumor phenotype. Clin. Cancer Res..

[32] Kiiski JI (2014). Exome sequencing identifies FANCM as a susceptibility gene for triple-negative breast cancer. Proc. Natl. Acad. Sci. USA.

[33] Peterlongo P (2015). FANCM c.5791C > T nonsense mutation (rs144567652) induces exon skipping, affects DNA repair activity and is a familial breast cancer risk factor. Hum. Mol. Genet..

[34] Dicks E (2017). Germline whole exome sequencing and large-scale replication identifies *FANCM* as a likely high grade serous ovarian cancer susceptibility gene. Oncotarget.

[35] Catucci I (2018). Individuals with FANCM biallelic mutations do not develop Fanconi anemia, but show risk for breast cancer, chemotherapy toxicity and may display chromosome fragility. Genet. Med..

[36] Bogliolo M (2018). Biallelic truncating FANCM mutations cause early-onset cancer but not Fanconi anemia. Genet. Med..

[37] Milne RL (2017). Identification of ten variants associated with risk of estrogen-receptor-negative breast cancer. Nat. Genet..

