# Supplementary material for: Two truncating variants in FANCC and breast cancer risk
Source: Sci Rep. 2019 Aug 29;9:12524. doi: 10.1038/s41598-019-48804-y (PMC6715680; doi:10.1038/s41598-019-48804-y)

# Two truncating variants in *FANCC* and breast cancer risk

Thilo Dörk^1^, Paolo Peterlongo^2^, Arto Mannermaa^3-5^, Manjeet K. Bolla^6^, Qin Wang^6^, Joe Dennis^6^, Thomas Ahearn^7^, Irene L. Andrulis^8, 9^, Hoda Anton-Culver^10^, Volker Arndt^11^, Kristan J. Aronson^12^, Annelie Augustinsson^13^, Laura E. Beane Freeman^7^, Matthias W. Beckmann^14^, Alicia Beeghly^15^, Sabine Behrens^16^, Marina Bermisheva^17^, Carl Blomqvist^18, 19^, Natalia V. Bogdanova^1, 20, 21^, Stig E. Bojesen^22-24^, Hiltrud Brauch^25-27^, Hermann Brenner^11, 27, 28^, Barbara Burwinkel^29, 30^, Federico Canzian^31^, Tsun L. Chan^32, 33^, Jenny Chang-Claude^16, 34^, Stephen J. Chanock^7^, Ji-Yeob Choi^35, 36^, Hans Christiansen^20^, Christine L. Clarke^37^, Fergus J. Couch^38^, Kamila Czene^39^, Mary B. Daly^40^, Isabel dos-Santos-Silva^41^, Miriam Dwek^42^, Diana M. Eccles^43^, Arif B. Ekici^44^, Mikael Eriksson^39^, D. Gareth Evans^45, 46^, Peter A. Fasching^14, 47^, Jonine Figueroa^7, 48, 49^, Henrik Flyger^50^, Lin Fritschi^51^, Marike Gabrielson^39^, Manuela Gago-Dominguez^52, 53^, Chi Gao^54, 55^, Susan M. Gapstur^56^, Montserrat García-Closas^7, 57^, José A. García-Sáenz^58^, Mia M. Gaudet^56^, Graham G. Giles^59-61^, Mark S. Goldberg^62, 63^, David E. Goldgar^64^, Pascal Guénel^65^, Lothar Haeberle^66^, Christopher A. Haiman^67^, Niclas Håkansson^68^, Per Hall^39, 69^, Ute Hamann^70^, Mikael Hartman^71, 72^, Jan Hauke^73-75^, Alexander Hein^14^, Peter Hillemanns^1^, Frans B.L. Hogervorst^76^, Maartje J. Hooning^77^, John L. Hopper^60^, Tony Howell^78^, Dezheng Huo^79^, Hidemi Ito^80, 81^, Motoki Iwasaki^82^, Anna Jakubowska^83, 84^, Wolfgang Janni^85^, Esther M. John^86^, Audrey Jung^16^, Rudolf Kaaks^16^, Daehee Kang^35, 36, 87^, Pooja Middha Kapoor^16, 88^, Elza Khusnutdinova^17, 89^, Sung-Won Kim^90^, Cari M. Kitahara^91^, Stella Koutros^7^, Peter Kraft^54, 55^, Vessela N. Kristensen^92, 93^, Ava Kwong^32, 94, 95^, Diether Lambrechts^96, 97^, Loic Le Marchand^98^, Jingmei Li^99^, Sara Lindström^100, 101^, Martha Linet^91^, Wing-Yee Lo^25, 26^, Jirong Long^15^, Artitaya Lophatananon^102, 103^, Jan Lubiński^83^, Mehdi Manoochehri^70^, Siranoush Manoukian^104^, Sara Margolin^69, 105^, Elena Martinez^53, 106^, Keitaro Matsuo^80, 81^, Dimitris Mavroudis^107^, Alfons Meindl^108^, Usha Menon^109^, Roger L. Milne^59, 60, 110^, Nur Aishah Mohd Taib^111^, Kenneth Muir^102, 103^, Anna Marie Mulligan^112, 113^, Susan L. Neuhausen^114^, Heli Nevanlinna^115^, Patrick Neven^116^, William G. Newman^45, 46^, Kenneth Offit^117, 118^, Olufunmilayo I. Olopade^79^, Andrew F. Olshan^119^, Janet E. Olson^120^, Håkan Olsson^13^, Sue K. Park^35, 36, 87^, Tjoung-Won Park-Simon^1^, Julian Peto^41^, Dijana Plaseska-Karanfilska^121^, Esther Pohl-Rescigno^73-75^, Nadege Presneau^42^, Brigitte Rack^85^, Paolo Radice^122^, Muhammad U. Rashid^70, 123^, Gad Rennert^124^, Hedy S. Rennert^124^, Atocha Romero^125^, Matthias Ruebner^66^, Emmanouil Saloustros^126^, Marjanka K. Schmidt^127, 128^, Rita K. Schmutzler^73-75^, Michael O. Schneider^66^, Minouk J. Schoemaker^129^, Christopher Scott^120^, Chen-Yang Shen^130, 131^, Xiao-Ou Shu^15^, Jacques Simard^132^, Susan Slager^120^, Snezhana Smichkoska^133^, Melissa C. Southey^110, 134^, John J. Spinelli^135, 136^, Jennifer Stone^60, 137^, Harald Surowy^29, 30^, Anthony J. Swerdlow^129, 138^, Rulla M. Tamimi^54, 55, 139^, William J. Tapper^140^, Soo H. Teo^111, 141^, Mary Beth Terry^142^, Amanda E. Toland^143^, Rob A.E.M. Tollenaar^144^, Diana Torres^70, 145^, Gabriela Torres-Mejía^146^, Melissa A. Troester^119^, Thérèse Truong^65^, Shoichiro Tsugane^147^, Michael Untch^148^, Celine M. Vachon^149^, Ans M.W. van den Ouweland^150^, Elke M. van Veen^45, 46^, Joseph Vijai^117, 118^, Camilla Wendt^105^, Alicja Wolk^68, 151^, Jyh-Cherng Yu^152^, Wei Zheng^15^, Argyrios Ziogas^10^, Elad Ziv^153^, ABCTB Investigators^154^, NBCS Collaborators^92, 93, 155-164^, Alison M. Dunning^165^, Paul D.P. Pharoah^6, 165^, Detlev Schindler^166^, Peter Devilee^167, 168^, Douglas F. Easton^6, 165^.

**Supplementary Table and Figure Legends:**

**Supplementary Table 1:** Overall and subgroup association analysis of *PALB2/FANCN* variant p.R414X with breast cancer risk. Results are given as odds ratios (OR) with 95% confidence interval (CI) and p-value (p). Cases in subgroups were compared to the frequency 3/49,825 in all controls. Familial cases were defined as those with a first-degree family history of breast cancer; premenopausal cases were those with age at diagnosis < 50 years. ER, estrogen-receptor; TNBC, triple-negative breast cancer.

**Supplementary Table 2:** *FANCC* and *PALB2/FANCN* mutation carriers listed per study that participated in the Oncoarray genotyping within the Breast Cancer Association Consortium. *Asian study with no participant of European descent.

**Supplementary Table 3:** Ethical review boards listed per study that participated in the Oncoarray genotyping within the Breast Cancer Association Consortium.

**Supplementary Figure 1:** Cluster plots showing Oncoarray genotyping results for the tested truncating variants in *FANCC* (upper panel) and *FANCN/PALB2* (bottom)

**Supplementary Table 1:** *FANCC* and *PALB2/FANCN* mutation carriers by study

| **Study** | ***FANCC**p.R185X** | | ***FANCC**p.R548X** | | ***PALB2**p.R414X** | |
| --- | --- | --- | --- | --- | --- | --- |
|  | **Carriers n** | **Total n** | **Carriers n** | **Total n** | **Carriers n** | **Total n** |
| **2SISTER** | 0 | 1,118 | 1 | 1,117 | 1 | 1,118 |
| **ABCFS** | 3 | 1,304 | 0 | 1,304 | 0 | 1,304 |
| **ABCS** | 0 | 536 | 0 | 536 | 1 | 536 |
| **ABCTB** | 2 | 1,328 | 0 | 1,328 | 0 | 1,328 |
| **ACP*** | 0 | 1,395 | 0 | 1,395 | 0 | 1,394 |
| **AHS** | 0 | 1,651 | 0 | 1,651 | 0 | 1,650 |
| **BBCC** | 0 | 664 | 0 | 664 | 0 | 664 |
| **BBCS** | 0 | 564 | 0 | 564 | 0 | 564 |
| **BCEES** | 0 | 1,618 | 0 | 1,618 | 0 | 1,618 |
| **BCFR-NY** | 0 | 481 | 0 | 481 | 0 | 481 |
| **BCFR-PA** | 0 | 139 | 0 | 139 | 0 | 139 |
| **BCFR-UTAH** | 0 | 102 | 0 | 102 | 0 | 102 |
| **BCINIS*** | 0 | 2,161 | 0 | 2,161 | 0 | 2,161 |
| **BREOGAN** | 0 | 2,102 | 1 | 2,102 | 2 | 2,101 |
| **BSUCH** | 0 | 444 | 0 | 445 | 0 | 445 |
| **CAMA*** | 0 | 1,365 | 0 | 1,366 | 1 | 1,366 |
| **CBCS** | 0 | 1,998 | 1 | 1,998 | 0 | 1,998 |
| **CCGP** | 0 | 1,004 | 0 | 1,004 | 0 | 1,004 |
| **CECILE** | 0 | 465 | 0 | 465 | 0 | 465 |
| **CGPS** | 2 | 2,125 | 0 | 2,127 | 0 | 2,127 |
| **COLBCCC** | 0 | 1,194 | 0 | 1,194 | 0 | 1,194 |
| **CPSII** | 1 | 6,081 | 2 | 6,081 | 1 | 6,081 |
| **CTS** | 1 | 1,766 | 1 | 1,766 | 0 | 1,766 |
| **DIETCOMPLYF** | 0 | 711 | 0 | 711 | 0 | 711 |
| **EPIC** | 1 | 7,489 | 1 | 7,491 | 3 | 7,491 |
| **ESTHER** | 0 | 483 | 0 | 483 | 0 | 483 |
| **FHRISK** | 0 | 442 | 0 | 442 | 0 | 442 |
| **GC-HBOC** | 0 | 5,226 | 2 | 5,227 | 4 | 5,227 |
| **GENICA** | 0 | 744 | 0 | 743 | 1 | 744 |
| **GEPARSIXTO** | 0 | 387 | 0 | 387 | 0 | 387 |
| **GESBC** | 0 | 539 | 0 | 539 | 0 | 539 |
| **HABCS** | 0 | 1,794 | 0 | 1,794 | 0 | 1,793 |
| **HCSC** | 0 | 426 | 0 | 426 | 0 | 426 |
| **HEBCS** | 0 | 458 | 0 | 457 | 0 | 458 |
| **HERPACC*** | 0 | 565 | 0 | 565 | 0 | 565 |
| **HKBCS*** | 0 | 1,018 | 0 | 1,018 | 0 | 1,018 |
| **HMBCS** | 0 | 461 | 0 | 461 | 0 | 461 |
| **HUBCS** | 0 | 331 | 0 | 331 | 1 | 331 |
| **KARBAC** | 1 | 503 | 0 | 503 | 0 | 503 |
| **KARMA** | 0 | 8,670 | 0 | 8,668 | 4 | 8,671 |
| **KBCP** | 0 | 801 | 0 | 800 | 0 | 801 |
| **KOHBRA*** | 0 | 2,129 | 0 | 2,129 | 0 | 2,129 |
| **LMBC** | 0 | 2,073 | 0 | 2,073 | 1 | 2,073 |
| **MABCS** | 0 | 181 | 0 | 182 | 0 | 181 |
| **MARIE** | 0 | 801 | 0 | 801 | 0 | 801 |
| **MBCSG** | 0 | 1,154 | 0 | 1,154 | 0 | 1,154 |
| **MCBCS** | 0 | 1,147 | 0 | 1,147 | 0 | 1,147 |
| **MCCS** | 1 | 2,028 | 0 | 2,028 | 0 | 2,028 |
| **MEC** | 3 | 2,767 | 0 | 2,767 | 0 | 2,767 |
| **MISS** | 0 | 2,246 | 0 | 2,245 | 0 | 2,245 |
| **MMHS** | 1 | 2,017 | 0 | 2,019 | 0 | 2,019 |
| **MSKCC** | 0 | 138 | 0 | 138 | 0 | 138 |
| **MTLGEBCS** | 0 | 511 | 0 | 511 | 0 | 511 |
| **MYBRCA*** | 0 | 2,103 | 0 | 2,103 | 0 | 2,103 |
| **NBCS** | 1 | 1,282 | 0 | 1,283 | 0 | 1,283 |
| **NBHS** | 1 | 1,680 | 1 | 1,682 | 0 | 1,682 |
| **NC-BCFR** | 0 | 1,680 | 0 | 1,682 | 1 | 1,681 |
| **NCBCS** | 0 | 6,039 | 1 | 6,042 | 1 | 6,042 |
| **NGOBCS*** | 0 | 735 | 0 | 735 | 0 | 735 |
| **NHS** | 0 | 3,394 | 0 | 3,393 | 0 | 3,394 |
| **NHS2** | 0 | 3,512 | 2 | 3,508 | 0 | 3,512 |
| **OFBCR** | 2 | 2,039 | 0 | 2,038 | 1 | 2,039 |
| **ORIGO** | 0 | 1,713 | 0 | 1,713 | 0 | 1,713 |
| **PBCS** | 0 | 3,976 | 0 | 3,976 | 0 | 3,974 |
| **PKARMA** | 0 | 882 | 0 | 882 | 0 | 882 |
| **PLCO** | 2 | 4,996 | 1 | 4,995 | 0 | 4,996 |
| **POSH** | 1 | 1,087 | 1 | 1,088 | 0 | 1,088 |
| **PREFACE** | 0 | 2,989 | 1 | 2,989 | 0 | 2,989 |
| **PROCAS** | 3 | 2,303 | 0 | 2,303 | 1 | 2,303 |
| **RBCS** | 0 | 714 | 0 | 714 | 1 | 714 |
| **SBCGS*** | 0 | 1,775 | 0 | 1,774 | 0 | 1,775 |
| **SEARCH** | 6 | 6,728 | 0 | 6,728 | 2 | 6,728 |
| **SEBCS*** | 0 | 2,209 | 0 | 2,210 | 0 | 2,210 |
| **SGBCC*** | 0 | 1,599 | 0 | 1,599 | 0 | 1,598 |
| **SISTER** | 5 | 3,898 | 2 | 3,897 | 0 | 3,898 |
| **SKKDKFZS** | 0 | 1,095 | 0 | 1,095 | 0 | 1,095 |
| **SMC** | 0 | 2,212 | 1 | 2,213 | 0 | 2,213 |
| **SUCCESSB** | 0 | 440 | 0 | 440 | 1 | 440 |
| **SUCCESSC** | 0 | 2,836 | 0 | 2,835 | 2 | 2,836 |
| **SZBCS** | 0 | 560 | 0 | 561 | 0 | 561 |
| **TNBCC** | 0 | 620 | 1 | 620 | 0 | 620 |
| **TWBCS*** | 0 | 807 | 0 | 806 | 0 | 807 |
| **UCIBCS** | 0 | 759 | 0 | 759 | 0 | 759 |
| **UKBGS** | 1 | 2,337 | 0 | 2,337 | 0 | 2,336 |
| **UKOPS** | 2 | 974 | 0 | 974 | 0 | 974 |
| **USRT** | 0 | 3,459 | 0 | 3,460 | 1 | 3,460 |
| **WAABCS** | 0 | 622 | 0 | 622 | 1 | 622 |
| **Total** | **40** | **153,899** | **20** | **153,904** | **32** | **153,912** |

**Supplementary Table 2:** Overall and stratified analysis of *PALB2/FANCN* variant p.R414X

| **Stratum** | **Cases** | **Controls** | **Odds Ratio (95% CI)** | **p** |
| --- | --- | --- | --- | --- |
| Overall | 22/ 64,780 | 3/ 49,825 | 5.89 (1.76; 19.74) | 0.004 |
| ER-ve | 10/ 10,132 |  | 18.97 (5.15; 69.85) | 9.4 x 10^-6^ |
| ER+ve | 8/ 40,882 |  | 3.65 (0.96; 13.87) | 0.058 |
| TNBC | 7/ 4,130 |  | 35.11 (8.89; 138.63) | 3.8 x 10^-7^ |
| Ductal | 18/ 36,714 |  | 8.41 (2.46; 28.73) | 0.001 |
| Lobular | 1/ 6,847 |  | 3.71 (0.37; 36.77) | 0.263 |
| High grade | 9/ 14,587 |  | 11.19 (2.97; 42.22) | 3.6 x 10^-4^ |
| Node-positive | 5/ 15,944 |  | 5.45 (1.25; 23.67) | 0.024 |
| Familial | 5/ 9,725 |  | 12.37 (2.84; 53.92) | 0.001 |
| Premenopausal | 11/ 22,254 |  | 8.48 (2.33; 30.84) | 0.001 |
| Bilateral | 2/ 2,742 |  | 19.43 (3.16; 119.27) | 0.001 |

**Supplementary Table 3:** List of ethical review boards per study

| **Acronym** | **Country** | **Approval Committee** |
| --- | --- | --- |
| 2SISTER | USA | Institutional Review Board of the National Institute of Environmental Health Sciences; NIH and the Copernicus Group Independent Review Board |
| ABCFS | Australia | The University of Melbourne Health Sciences Human Ethics Sub-Committee (HESC) |
| ABCS | Netherlands | Leiden University Medical Center (LUMC) Commissie Medische Ethiek and Protocol Toetsingscommissie van het Nederlands Kanker Instituut/Antoni van Leeuwenhoek Ziekenhuis |
| ABCTB | Australia | The Queenland Institute of Medical Research Human Research Ethics Committee (QIMR-HREC) |
| ACP | Thailand | Ethics Committee of National Cancer Institute Thailand; Prince of Songkla University Faculty of Medicine Ethics Committee; Khon Kaen University Ethics Committee for Human Research; HRH Princess Maha Chakri Sirindhorn Medical Centre (MSMC) Ethics Committee |
| AHS | USA | National Institute of Health (NIH) IRB |
| BBCC | Germany | Friedrich-Alexander-Universitat Erlangen-Nurnberg Medizinische Fakultat Ethik-Commission |
| BBCS | UK | South East Multi-Centre Research Ethics Committee |
| BCEES | Australia | Government of Western Australia, Department of Health, Human Research Ethics Committee |
| BCFR-NY | USA | Columbia University Medical Center Institutional Review Board |
| BCFR-PA | USA | Institutional Review Board Fox Chase Cancer Center |
| BCFR-UTAH | USA | Institutional Review Board University of Utah |
| BCINIS | Israel | Carmel Medical Center |
| BREOGAN | Spain | Comité Autonómico de Ética de la Investigación de Galicia |
| BSUCH | Germany | Medizinische Fakultat Heidelberg Ethikkommission |
| CAMA | Mexico | IRB of the National Institute of Public Health (INSP) of Mexico; UCSF Committee on Human Research |
| CBCS | Canada | University of British Columbia - British Columbia Cancer Agency Research Ethics Board;  Queen’s University Human Ethics Committee |
| CCGP | Greece | Epistimoniko Symvoulio |
| CECILE | France | Comite Consultatif de Protection des Personnes dans la Recherche Biomedicale de Bicetre |
| CGPS | Denmark | Kobenhavns Amt den Videnskabsetiske Komite |
| COLBCCC | Colombia | Comite de Investigaciones y Etica Pontificia Universidad Javeriana |
| CPSII | USA | Emory University Institutional Review Board |
| CTS | USA | UC Irvine: Office of Research Institutional Review Board |
| DIETCOMPLYF | UK | The University College London Hospitals Ethics Committee |
| EPIC | Various within EU | Institutional Review Board of the International Agency for Research on Cancer, Lyon, France; Ethics Commission of the Faculty of Medicine of the University of Heidelberg . |
| ESTHER | Germany | Ruprecht-Karls-Universitat Medizinische Fakultat Heidelberg Ethikkommission |
| FHRISK | UK | NRES Committee North West - Greater Manchester Central |
| GC-HBOC | Germany | Ethik-Kommission der Medizinischen Fakultat der Universitat zu Koln |
| GENICA | Germany | Rheinische Friedrich-Wilhelms-Universitat Medizinische Einrichtungen Ethik-Kommission |
| GEPARSIXTO | Germany | Ethikkommission der Ärztkammer Nordrhein |
| GESBC | Germany | Ruprecht-Karls-Universitat Medizinische Fakultat Heidelberg Ethikkommission |
| HABCS | Germany | Medizinische Hochschule Hannover Ethik-Kommission |
| HCSC | Spain | Hospital Clínico San Carlos Ethical Committee |
| HEBCS | Finland | Helsingin ja uudenmaan sairaanhoitopiiri (Helsinki University Central Hospital Ethics Committee) |
| HERPACC | Japan | Ethics Committee for Human Genome Study at Aichi Cancer Center |
| HKBCS | Hong Kong | Institutional Review Board of the University of Hong Kong/Hospital Authority Hong Kong Cluster (HKU/HA HKW IRB) |
| HMBCS | Belarus | Medizinische Hochschule Hannover Ethik-Kommission |
| HUBCS | Russia | Ethical Committee of Institute of Biochemistry and Genetics Ufa Science Center |
| KARBAC | Sweden | Lokala Forskningsetikkommitten Nord |
| KARMA | Sweden | Regionala Etikprovningsnamnden i Stockholm (Regional Ethical Review Board in Stockholm) |
| KBCP | Finland | Pohjois-Savon Sairraanhoitopiirin Kuntayhtyma Tutkimuseettinen Toimikunta |
| KOHBRA | Korea | Seoul National University College of Medicine/Seoul National University Hospital Institutional Review Board (SNUCM/SNUH IRB) |
| LMBC | Belgium | Commissie Medische Ethiek van de Universitaire Ziekenhuizen Kuleuven |
| MABCS | Republic of North Macedonia | Ethic Subcommittee of Medicine, Pharmacy, Veterinary Medicine and Dentistry, Macedonian Academy of Sciences and Arts |
| MARIE | Germany | Ruprecht-Karls-Universitat Medizinische Fakultat Heidelberg Ethikkommission |
| MBCSG | Italy | Comitato Etico Indipendente della Fondazione IRCCS "Istituto Nazionale dei Tumori" |
| MCBCS | USA | Mayo Clinic IRB |
| MCCS | Australia | The Cancer Council Victoria Human Research Ethics Committee |
| MEC | USA | University of Southern California Health Sciences Campus IRB |
| MISS | Sweden | Regional Ethical Board in South Sweden |
| MMHS | USA | Mayo Clinic IRB |
| MSKCC | USA | Memorial Sloan-Kettering Cancer Center IRB |
| MTLGEBCS | Canada | McGill University IRB |
| MYBRCA | Malaysia | University Malaya Medical Centre Medical Ethics Committee |
| NBCS | Norway | Regional Komite for Medisinsk Forskningsetikk (Helseregion III Universitetet I Bergen, Universitetet I Oslo, Helseregion Sor, Helseregion II, and Ost-Norge) |
| NBHS | USA | Vanderbilt University IRB |
| NC-BCFR | USA | Cancer Prevention Institute of California IRB |
| NCBCS | USA | Office of Human Research Ethics, the University of North Carolina, Chapel Hill |
| NGOBCS | Japan | Institutional review board of the National Cancer Center, Tokyo. |
| NHS | USA | Brigham and Women’s Hospital IRB; Harvard T.H. Chan School of Public Health IRB |
| NHS2 | USA | Brigham and Women’s Hospital IRB; Harvard T.H. Chan School of Public Health IRB |
| OFBCR | Canada | Mount Sinai Hospital Research Ethics Board |
| ORIGO | Netherlands | Medical Ethical Committee and Board of Directors of the Leiden University Medical Center (LUMC) |
| PBCS | Poland | National Institute of Health (NIH) IRB |
| PKARMA | Sweden | Regionala Etikprovningsnamnden i Stockholm (Regional Ethical Review Board in Stockholm) |
| PLCO | USA | National Cancer Institute Special Studies Institutional Review Board (NCI-SSIRB) |
| POSH | UK | South West Multi-centre Research Ethics Committee |
| PREFACE | Germany | Friedrich-Alexander-Universitat Erlangen-Nurnberg Medizinische Fakultat Ethik-Commission |
| PROCAS | UK | NRES Committee North West - Greater Manchester Central |
| RBCS | Netherlands | Medische Ethische Toetsings Commissie Erasmus Medisch Centrum |
| SBCGS | China | Vanderbilt University Medical Center IRB, Shanghai Cancer Institute IRB, and Shanghai Center for Disease Prevention and Control IRB |
| SEARCH | UK | Multi Centre Research Ethics Committee (MREC) |
| SEBCS | Korea | Seoul National University College of Medicine/Seoul National University Hospital IRB |
| SGBCC | Singapore | National Health Group (NHG) Domain Specific Review Board (DSRB) |
| SISTER | USA | Institutional Review Board of the National Institute of Environmental Health Sciences; NIH and the Copernicus Group Independent Review Board |
| SKKDKFZS | Germany | Ethics Committee of the Medical Faculty Heidelberg |
| SMC | Sweden | Regionala Etikprovningsnamnden i Stockholm (Regional Ethical Review Board in Stockholm) |
| SUCCESSB | Germany | Ethikkommission der Medizinischen Fakultät der Ludwig-Maximilians-Universität München |
| SUCCESSC | Germany | Ethikkommission der Medizinischen Fakultät der Heinrich Heine Universität Düsseldorf |
| SZBCS | Poland | Komisji Bioetycznej Pomorskiej Akademii Medycznej |
| TNBCC | Various | Mayo Clinic IRB |
| TWBCS | Taiwan | Human Subject Research Ethics Committee/IRB Academia Sinica |
| UCIBCS | USA | UC Irvine: Office of Research Institutional Review Board |
| UKBGS | UK | South East Multi-Centre Research Ethics Committee |
| UKOPS | UK | NRES Committee London – Harrow |
| USRT | USA | National Institute of Health (NIH) IRB |
| WAABCS | Nigeria, Cameroon & Uganda | The University of Chicago IRB; Le Comite National d'Ethique de la Recherche pour la Sante Humaine (CNERSH) and Makerere University Research Ethics Committee |

# Supplementary Figure 1


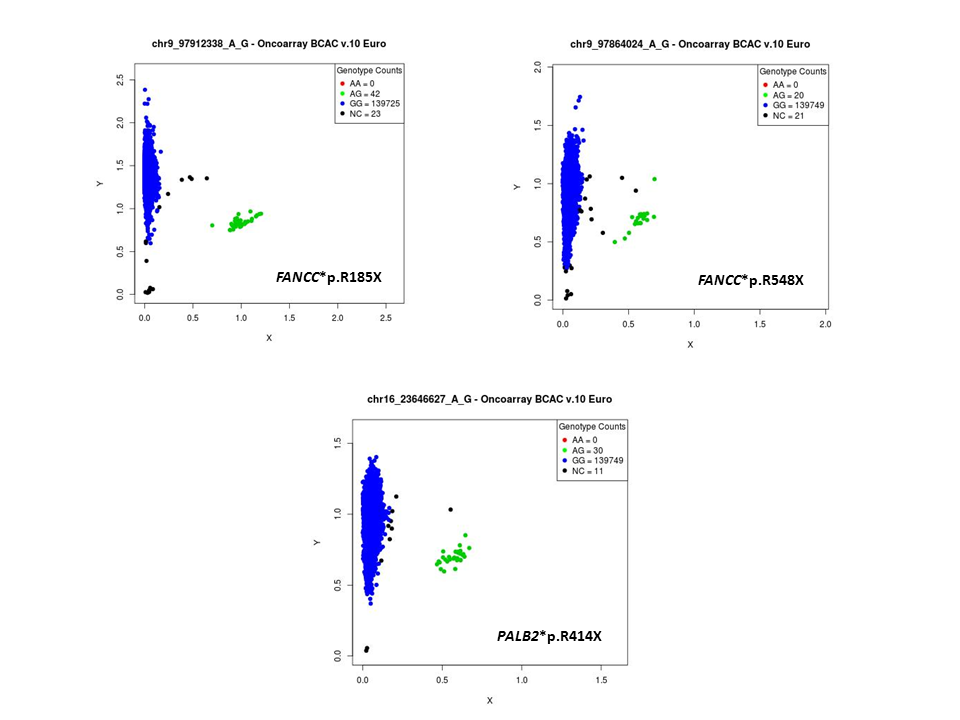

Supplement: Supplementary file 1 — Supplemental Info [file 41598_2019_48804_MOESM1_ESM.docx]
